# Perinatal care in SARS-CoV-2 infected women: the lesson learnt from a national prospective cohort study during the pandemic in Italy

**DOI:** 10.1186/s12889-023-17390-0

**Published:** 2023-12-21

**Authors:** Edoardo Corsi Decenti, Michele Antonio Salvatore, Donatella Mandolini, Letizia Sampaolo, Paola D’Aloja, the Italian Obstetric Surveillance System COVID-19 Consortium, Irene Alberi, Saverio Arena, Roberto Brunelli, Angelo Cagnacci, Franco Camandona, Paola Casucci, Sebastiano Caudullo, Irene Cetin, Marcello Ceccaroni, Andrea Ciavattini, Antonella Cromi, Pietro Dal Rì, Lidia Di Cerbo, Francesca Di Sebastiano, Daniele Farsetti, Massimo Piergiuseppe Franchi, Enrico Iurlaro, Livio Leo, Marco Liberati, Lucia Li Sacchi, Stefania Livio, Mariavittoria Locci, Massimo Lovotti, Luca Marozio, Claudio Martini, Gianpaolo Maso, Federico Mecacci, Alessandra Meloni, Anna Domenica Mignuoli, Luisa Mondo, Danila Morano, Luisa Patanè, Rocco Paradiso, Antonio Pellegrino, Francesca Perotti, Enrica Perrone, Roberta Piccino, Federico Prefumo, Luca Ramenghi, Morena Rocca, Alessia Sala, Marina Sangaletti, Valeria Savasi, Sergio Crescenzo Antonio Schettini, Daniela Simeone, Serena Simeone, Martin Steinkasserer, Fabrizio Taddei, Marina Tesorone, Vito Trojano, Caterina Tronci, Micaela Veneziano, Patrizia Vergani, Antonella Vimercati, Serena Donati

**Affiliations:** National Centre for Disease Prevention and Health Promotion, Istituto Superiore Di Sanità - Italian National Institute of Health, Viale Regina Elena 299, 00161 Rome, Italy

**Keywords:** COVID-19, SARS-CoV-2, Pregnancy, Childbirth care, Perinatal care, Skin-to-skin, Rooming-in, Breastfeeding

## Abstract

**Background:**

Despite the growing importance given to ensuring high-quality childbirth, perinatal good practices have been rapidly disrupted by SARS-CoV-2 pandemic. This study aimed at describing the childbirth care provided to infected women during two years of COVID-19 emergency in Italy.

**Methods:**

A prospective cohort study enrolling all women who gave birth with a confirmed SARS-CoV-2 infection within 7 days from hospital admission in the 218 maternity units active in Italy during the periods February 25, 2020-June 30, 2021, and January 1-May 31, 2022. Perinatal care was assessed by evaluating the prevalence of the following indicators during the pandemic: presence of a labour companion; skin-to-skin; no mother–child separation at birth; rooming-in; breastfeeding. Logistic regression models including women’ socio-demographic, obstetric and medical characteristics, were used to assess the association between the adherence to perinatal practices and different pandemic phases.

**Results:**

During the study period, 5,360 SARS-CoV-2 positive women were enrolled. Overall, among those who had a vaginal delivery (*n* = 3,574; 66.8%), 37.5% had a labour companion, 70.5% of newborns were not separated from their mothers at birth, 88.1% were roomed-in, and 88.0% breastfed. These four indicators showed similar variations in the study period with a negative peak between September 2020 and January 2021 and a gradual increase during the Alpha and Omicron waves. Skin-to-skin (mean value 66.2%) had its lowest level at the beginning of the pandemic and gradually increased throughout the study period. Among women who had a caesarean section (*n* = 1,777; 33.2%), all the indicators showed notably worse outcomes with similar variations in the study period. Multiple logistic regression analyses confirm the observed variations during the pandemic and show a lower adherence to good practices in southern regions and in maternity units with a higher annual number of births.

**Conclusions:**

Despite the rising trend in the studied indicators, we observed concerning substandard childbirth care during the SARS-CoV-2 pandemic. Continued efforts are necessary to underscore the significance of the experience of care as a vital component in enhancing the quality of family-centred care policies.

**Supplementary Information:**

The online version contains supplementary material available at 10.1186/s12889-023-17390-0.

## Introduction

As highlighted by the World Health Organization (WHO), global agendas are expanding their focus to ensure that the approximately 140 million women who give birth annually without risk factors not only survive possible labour complications but also thrive and reach their full potential for health and life [1]. Although WHO recommends high-quality labour and childbirth care with a focus on improved woman-centred outcomes, provision of labour companions, support of breastfeeding through skin-to-skin contact and rooming-in are not consistently prioritized in many settings [1]. These family-centred care policies are inexpensive and essential components of the care experience, proven to improve perinatal outcomes and childbirth satisfaction [2–4]. Achieving appropriate perinatal care represents an undelayable and challenging global goal and the impact of the SARS-CoV-2 pandemic needs to be examined in depth. Though, after years of effort, the COVID-19 emergency has negatively affected the adherence to good practices such as rooming-in, skin-to-skin and companionship during labour and childbirth [5, 6]. Parental experiences highlighted how maternity care during the pandemic did not adhere to WHO standards of quality maternity care [1, 7]. They also showed how crucial it is for healthcare institutions to continuously appraise the implementation of restrictive practices that diverge from evidence-based frameworks underpinning quality care [4, 7].

In Italy, from February 25, 2020, embracing with the preparedness measures recommended by the European Centre for Disease Prevention and Control [8], the Italian Obstetric Surveillance System of the *Istituto Superiore di Sanità* (Italian National Institute of Health, INIH) enrolled all pregnant women who tested positive for SARS-CoV-2 to study the impact of the virus on pregnancy and childbirth throughout the pandemic period [9, 10].

This study aimed to describe the perinatal care provided to infected women during the SARS-CoV-2 pandemic in Italy, updating data collected at the beginning of the emergency [11].

## Methods

This prospective cohort study [9] includes all pregnant women who gave birth with a confirmed SARS-CoV-2 infection within 7 days from hospital admission between February 25, 2020, and June 30, 2021, and between January 1, 2022, and May 31, 2022. Due to organisational issues, the data collection was stopped between July 1, 2021, and December 31, 2021.

Confirmed SARS-CoV-2 infection was determined by detecting viral RNA from nasopharyngeal swab. In Italy, from May 2020, all pregnant women admitted to hospital were tested for SARS-CoV-2, regardless of symptoms or exposure.

Detailed information on maternal socio-demographic characteristics, medical and obstetric history, disease management, and maternal and perinatal outcomes for all the eligible cases was notified by trained clinicians through an online form. This information was transmitted encrypted to the INIH through a dedicated server by the clinicians of the 218 participating maternity units (Appendix in Additional file 1).

Perinatal care was assessed using the following indicators, considered in the present study as dichotomous outcome variables:

(i) presence of a labour companion;
(ii) skin-to-skin;
(iii) no mother–child separation at birth;
(iv) rooming-in;
(v) breastfeeding.

The pandemic phases were considered as the exposure variable and grouped according to the predominant SARS-CoV-2 circulating variant and the adopted health policies (INIH 2023):

(i) February 25, 2020 – May 31, 2020: wild-type virus, phase 1;
(ii) June 1, 2020 – August 31, 2020: wild-type virus, phase 2;
(iii) September 1, 2020 – January 31, 2021: wild-type virus, phase 3;
(iv) February 1, 2021 – June 30, 2021: Alpha variant;
(v) January 1, 2022– May 31, 2022: Omicron variant.

Maternal age (< 30, 30–34, ≥ 35 years), citizenship (Italian, not Italian), educational level (low, primary school or lower; medium, high school; high, bachelor’s degree or higher), parity (nulliparous, multiparous), presence/absence of diagnosed COVID-19 pneumonia, gestational age at birth (≤ 31 weeks, 32–36 weeks, ≥ 37 weeks), geographical area (North, Centre, and South Italy), and the annual number of deliveries of the maternity unit (< 1000, 1000–1999, ≥ 2000) represent potential risk factors for adherence to good practices, as suggested by previous studies [12, 13], and were included in the analysis as potential confounders. Limited to the period of the Omicron variant, when the vaccination policy in Italy was well-established, vaccine protection was also considered. According to a previously published article [10], the vaccine protection classes were categorized as follows:

(i) women who received at least one dose during pregnancy and those who completed the vaccine cycle with the first booster were considered protected against moderate (confirmed pneumonia requiring at most oxygen therapy) or severe (confirmed pneumonia requiring mechanical ventilatory support and/or intensive care unit admission) COVID-19;
(ii) unvaccinated women and those who received one or two doses before pregnancy and were SARS-CoV-2 positive ≥ 22 weeks of gestation were considered unprotected;
(iii) women with missing vaccination information and those who received one or two doses before pregnancy and were SARS-CoV-2 positive < 22 weeks of gestation were categorized as ‘unknown with regard to protection status’.

Statistical analyses were carried out using STATA/MP version 15. Frequency distributions, prevalence and odds ratios (ORs) with their 95% confidence intervals (CI) were used to describe data. Percentages were calculated based on cases with available information. Frequency distributions by socio-demographic, obstetric, and medical characteristics were computed for women with vaginal delivery or caesarean section (CS). Pearson’s Chi-squared test assessed significant differences between the two groups. The prevalence of outcome variables in the five pandemic phases was computed stratifying by mode of delivery. The association between the outcome variables and the pandemic phases for both vaginal births and CSs was assessed through multiple logistic regression models. These models estimated ORs adjusted for women’s socio-demographic, medical, and obstetric characteristics to evaluate the statistical significance of the changes in perinatal care in the study period. The models were applied to multiple-imputed data assuming data were missing at random. For each model, the imputation of 20 data sets was performed using chained equations [14]; Rubin’s rules were used to combine models estimates across the 20 data sets [15]. Additional models were also performed using complete cases only (i.e., by excluding records with missing information). The stepwise procedure (with a significance level of 0.05) was used to perform both models on imputed and non-imputed data.

## Results

During the study period, 5,360 women who gave birth with a confirmed SARS-CoV-2 infection within 7 days from hospital admission were notified. Among them, 3,574 (66.8%) had a vaginal delivery, while 1,777 (33.2%) underwent a CS (Table 1). The distribution of cases by pandemic phase, showed 2,212 cases (41.3%) during the three wild-type virus phases, 644 (12.0%) during the Alpha variant, and 2,504 (46.7%) during the Omicron variant phase. Compared to women who had a vaginal delivery, those who had a CS were more often aged ≥ 35 years (38.0% vs 29.5%; *p* < 0.001), affected by COVID-19 pneumonia (8.8% *vs* 3.1%; *p* < 0.001), and delivered more frequently preterm (19.2% *vs* 5.4%; *p* < 0.001). Epidural analgesia was required in 20.8% of vaginal deliveries.

**Table 1:** SARS-CoV-2 positive women characteristics by mode of delivery

	**Vaginal deliveries**		**Caesarean sections**		Total^*^		***p*-value**
	***n* = 3,574**		***n* = 1,777**		***N* = 5,360**		
	**n**	**%**	**n**	**%**	**n**	**%**	
Pandemic phase							
Wild-type virus, phase 1 (25 February—May 31, 2020)	202	5.7	103	5.8	307	5.7	*p* = 0.692
Wild-type virus, phase 2 (June 1—August 31, 2020)	78	2.2	39	2.2	117	2.2	
Wild-type virus, phase 3 (September 1, 2020 – January 31, 2021)	1,175	32.9	607	34.2	1788	33.4	
Alpha variant (February 1—June 30, 2021)	422	11.8	222	12.5	644	12.0	
Omicron variant (January 1—May 31, 2022)	1,697	47.5	806	45.4	2504	46.7	
Age							
< 30 years	1,279	36.4	506	28.8	1,789	33.9	*p* < 0.001
30–34 years	1,200	34.1	582	33.2	1,787	33.9	
≥ 35 years	1,035	29.5	667	38.0	1,702	32.2	
* missing*	*60*	*1.7*	*22*	*1.2*	*82*	*1.5*	
Citizenship							
Not Italian	934	26.1	439	24.7	1,373	25.6	*p* = 0.260
Italian	2,640	73.9	1,338	75.3	3,987	74.4	
Level of education							
Low	810	29.7	365	28.9	1,177	29.5	*p* = 0.357
Medium	1,238	45.5	604	47.8	1,843	46.2	
High	675	24.8	294	23.3	969	24.3	
* missing*	*851*	*23.8*	*514*	*28.9*	*1,371*	*25.6*	
Parity							
Nulliparous	1,571	44.6	752	43.4	2,329	44.3	
Multiparous	1,948	55.4	979	56.6	2,930	55.7	*p* = 0.410
* missing*	*55*	*1.5*	*46*	*2.6*	*101*	*1.9*	
Gestational age at birth							
≤ 31 weeks	17	0.5	83	4.7	101	1.9	*p* < 0.001
32–36 weeks	173	4.9	255	14.5	428	8.1	
≥ 37 weeks	3,351	94.6	1,425	80.8	4,777	90.0	
* missing*	*33*	*0.9*	*14*	*0.8*	*54*	*1.0*	
COVID-19 pneumonia							
No	3,463	96.9	1,621	91.2	5,091	95.0	*p* < 0.001
Yes	111	3.1	156	8.8	269	5.0	
Peridural anaesthesia in vaginal deliveries							
No	2,463	79.2					
Yes	645	20.8					
* missing*	*466*	*13.0*					
Type of caesarean section							
Elective			778	43.8			
Urgent/emergency due to maternal/foetal indication			934	52.6			
Urgent/emergency due to COVID-19			65	3.7			
Volume of deliveries of maternity units							
< 1,000	981	28.0	461	26.5	1,444	27.5	*p* = 0.381
1,000–1,999	1,264	36.0	621	35.7	1,888	35.9	
≥ 2,000	1,263	36.0	657	37.8	1,924	36.6	
* missing*	*66*	*1.8*	*38*	*2.1*	*104*	*1.9*	
Geographical location of maternity units							
North Italy	2,406	67.3	978	55.0	3,389	63.2	*p* < 0.001
Centre Italy	483	13.5	235	13.2	718	13.4	
South Italy	685	19.2	564	31.7	1,253	23.4	

Percentages were calculated based on cases with known information

^*^9 women with unknown information on mode of delivery

**Fig. 1:**
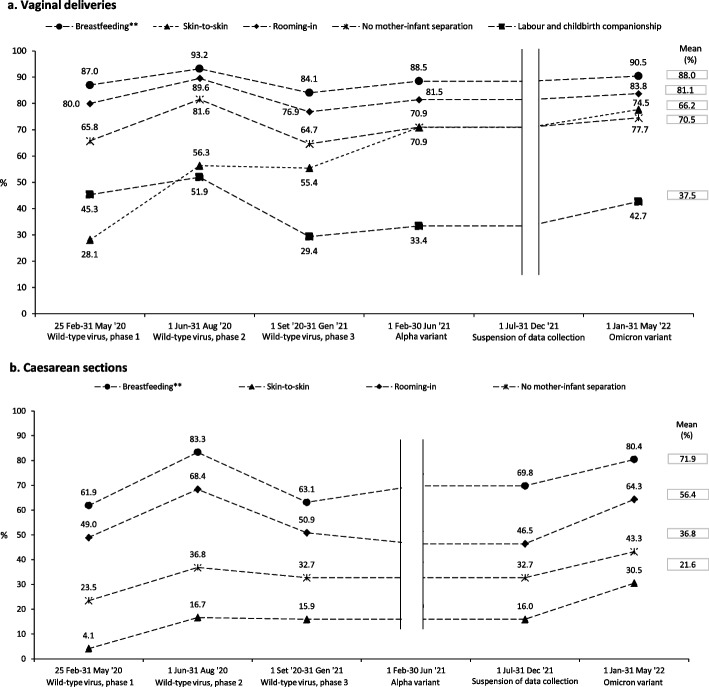
**a** Perinatal care offered to SARS-CoV-2 positive women who had a vaginal birth during the COVID-19 pandemic in Italy. **b** Perinatal care offered to SARS-CoV-2 positive women who underwent caesarean section during the COVID-19 pandemic in Italy. Labour and childbirth companionship prevalences are calulated for vaginal deliveries (*n* = 3,574). All the other indicators prevalences are calculated among live births in vaginal deliveries (*n* = 3,556) and caesarean section (*n* = 1,860). Prevalences are calculated by escluding cases with missing information. *Direct or pumped maternal breastmilk

Among vaginal deliveries, 37.5% of women had a labour companion. A decrease in percentage was observed with a negative peak during phase 3 of the wild-type virus (29.4%), followed by slight increase in subsequent phases (Fig. 1a). Among neonates born by vaginal delivery (*n* = 3,556 livebirths), 70.5% were not separated from their mothers at birth, 88.1% were roomed-in, and 88.0% were breastfed. These indicators showed the same trend as that recorded for the labour companion, with lower values during the third phase of the wild-type virus and a gradual increase during the Alpha and Omicron phases. Among neonates born by CS (*n* = 1,860 livebirths), those not separated from their mothers ranged from 23.5% to 43.3% (mean 36.8%), rooming-in from 49.0% to 64.3% (mean 56.4%), and breastfeeding from 61.9% to 80.4% (Fig. 1b). Skin-to-skin contact was practised for 28.1% of children born by vaginal delivery in the first phase of the pandemic and increased over the study period, peaking at 77.7% in the Omicron phase (Fig. 1a). For CS, skin-to-skin increased from 4.1% to 30.5% (Fig. 1b).

Table S1a and b, respectively for vaginal births and CSs, show frequency distributions used to calculate the prevalence and the number of missing values for all the indicators.

**Fig. 2:**
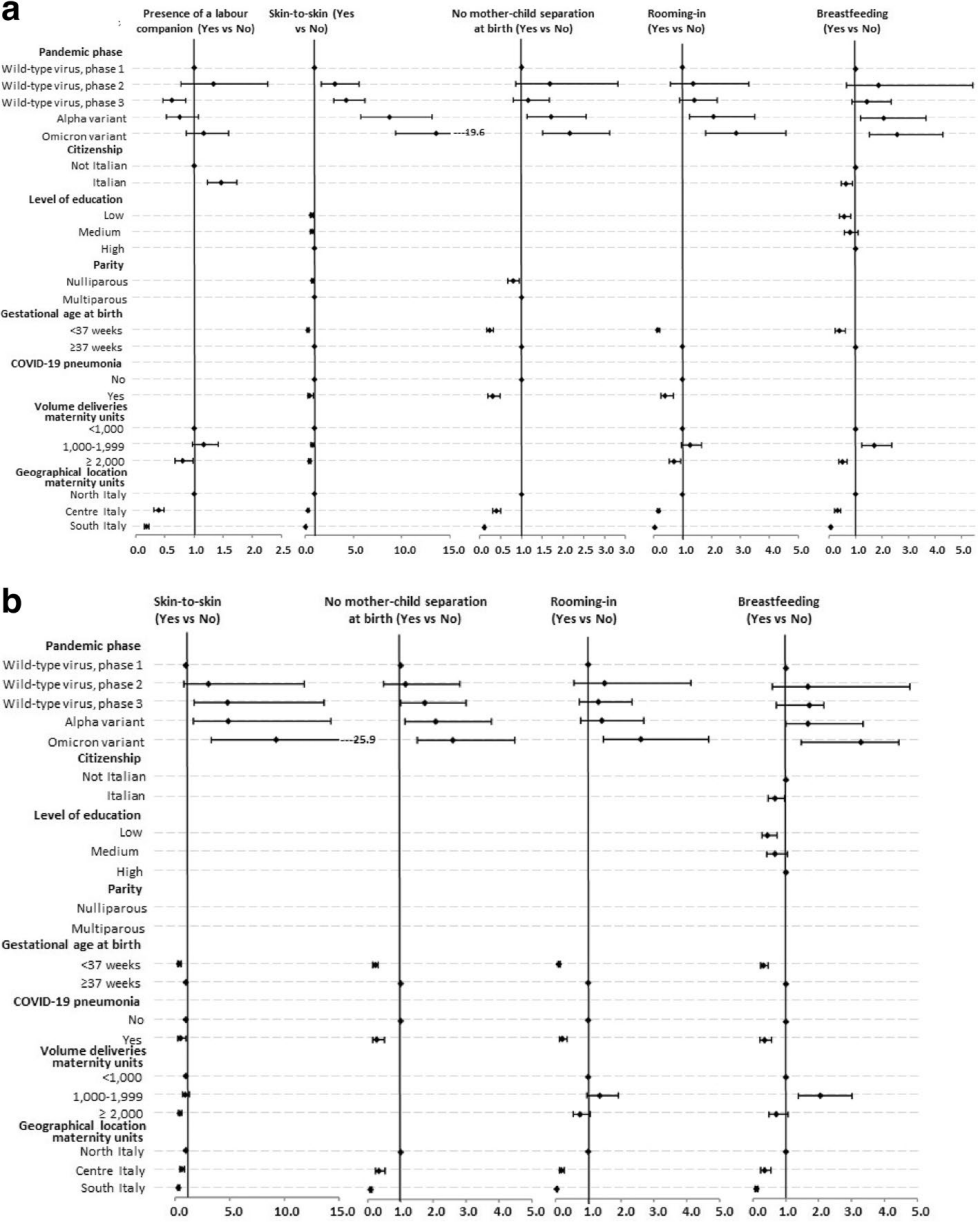
a Mutually adjusted odds ratios for the reported variables and 95% confidence intervals among women who had a vaginal birth. Logistic regression models on imputed data. b Mutually adjusted odds ratios for the reported variables and 95% confidence intervals among women who underwent caesarean section. Logistic regression models on imputed data. In the models performed by stepwise procedure, the following variables were considered: pandemic phase, age, citizenship, level of education, parity, gestational age at birth, COVID-19 pneumonia, volume of deliveries of maternity units, geographical location of maternity units

Among women who had a vaginal birth, there were significantly higher odds of no mother–child separation, rooming-in, and breastfeeding during the Alpha and Omicron waves compared to the odds in the first phases (Fig. 2a, Table S2a). Skin-to-skin odds were significantly higher in all subsequent phases following the first one. A similar pattern with minor variations was observed among women undergoing CSs (Fig. 2b, Table S2b). The presence of a labour companion during vaginal births showed a statistically significant decrease in occurrence in the third phase of the wild-type virus (OR 0.62, 95%CI 0.46–0.86) (Fig. 2a, Table S2a).

Among the other variables considered in the model, Italian women had significantly lower breastfeeding odds than foreigners (vaginal births: OR 0.63, 95%CI 0.45–0.88; CSs: OR 0.67, 95% CI: 0.46–0.97), while Italian women showed higher odds of having a labour companion than non-Italians (OR 1.47, 95%CI 1.23–1.74) (Fig. 2, Table S2). Among women with lower level of education, significantly lower odds were observed for breastfeeding (vaginal births: OR 0.56, 95%CI 0.39–0.81; CSs: OR 0.44, 95%CI 0.27–0.74) and, in vaginal births, for skin-to-skin contact (OR 0.72, 95%CI 0.56–0.92).

Lower odds were recorded for all the indicators in central and southern regions compared to northern regions in both vaginal deliveries and CSs. Maternity units with a higher annual number of deliveries (≥ 2,000) presented a lower odd for skin-to-skin and, in case of vaginal births, also for rooming-in and breastfeeding. With the exception of for labour companion and breastfeeding among vaginal births, a lower occurrence of all practices was recorded in the presence of COVID-19 pneumonia, and, except for labour companion, in case of preterm birth.

The results obtained by using only complete cases did not show significantly changes among both vaginal births (Table S3a) and CSs (Table S3-b).

The results of logistic regression models, based on multiple-imputed data related only to the Omicron phase, revealed that, compared to women protected by vaccination, those unvaccinated presented lower odds (adjusted for age, citizenship, level of education, parity, gestational age at birth, presence/absence of COVID-19 pneumonia, annual number of deliveries of the maternity unit, geographical area) of presence of having a labour companion (OR 0.72, 95%CI 0.55–0.95), skin-to-skin contact (OR 0.75, 95%CI 0.56–1.02), and rooming-in (OR 0.63, 95%CI 0.44–0.94) in vaginal births and lower odds of no mother–child separation (OR 0.66, 95%CI 0.45–0.98) and rooming-in (OR 0.63, 95%CI 0.41–0.96) in CSs (data not shown).

## Discussion

In this large cohort study, we aimed to provide a comprehensive description of childbirth care during the pandemic in Italy. We prospectively enrolled more than five thousand SARS-CoV-2 positive pregnant women at hospital admission. Despite the overall high-quality care provided to pregnant women in terms of clinical management and treatment and the low rate of CS compared to other similar European countries [16, 17], we recorded a substantial worsening of the family-centred practices during and after birth, especially in the southern Regions. The beginning of the pandemic emergency led to a deterioration of all the indicators of interest, with a slight improvement starting from the period of the SARS-CoV-2 Alpha variant. The studied trends show a slow adaptation of Italian maternity units and highlight organisational challenges in guaranteeing a respectful birth experience to all families.

During the study period, among women who had a vaginal delivery, less than four out of ten had a labour companion, ranging from 51.9% during the first pandemic summer to 29.4% in the following autumn. The IMAgiNE EURO online survey conducted in 12 countries of the WHO European Region from March 2020 to March 2021 showed that, on average, only 32% of women who underwent labour (*n* = 18,063) had a companion of choice [13]; the data from Italy was even lower than the average (21.5%). This discrepancy could be due to the different methodologies adopted, such as a voluntary online survey directly compiled by women *vs* data prospectively collected by clinicians. However, it shows how restrictive policies affected not only SARS-CoV-2 positive women but all those who gave birth during the pandemic. Unfortunately, there is a scarcity of pre-pandemic data about the topic. Although the Italian Certificate of childbirth assistance collects information about labour companionship, the presence of missing information in many records does not allow a reliable comparison [18]. In a recent systematic review including 77 studies from 27 countries (among these, 8 high-income countries) published between 2010 and 2021, one-third of the studies reported a labour companionship coverage below 40%, another one-third between 40–80%, and thirty-nine percent of studies an 80–100% coverage [19]. In the multiple logistic regression analysis, maternal citizenship appears to be associated with labour companionship, with an odds among Italian women 47% higher than foreign women (OR 1.47, 95% CI 1.23–1.74). For foreign women, the role of the labour companion, mainly the partner, is critical during the hospital stay, merely because they usually have better language proficiency. Therefore, this result can be considered unexpected and confirms the evidence that inequalities in childbirth exist because of different ethnic backgrounds [20]. In Italy, like in other countries, foreign women have lower access to antenatal care than Italian women, with fewer visits during pregnancy and the first contact more often postponed beyond the 10th week [21, 22]. On the contrary, Italian women showed lower occurrence of breastfeeding (OR 0.61, 95%CI 0.43–0.87). Previous surveys demonstrated that immigrant women, probably due to the different value of breastfeeding established in the country of origin, were more likely than non‐immigrants to initiate and maintain any breastfeeding [22–32]. Breastfeeding was also found to be less frequent among less educated women compared to more educated (vaginal births: OR 0.56, 95% CI 0.39-0.81; CS: OR 0.44, 95% 0.27-0.74) and so it was for the skin-to-skin contact among vaginal births (OR 0.72, 95% CI: 0.56-0.92). Previous studies reported that a low maternal level of education relates to poorer childbirth care, including the presence of labour companions and initiation of breastfeeding [22, 25, 26]. In the present study the occurrence of labour companionship was lower in maternity units with a higher volume of births (OR 0.81 95%CI 0.67–0.99), as well as the rooming-in practice (OR 0.70, 95% CI 0.53-0.93 among vaginal births), skin-to-skin (vaginal births: OR 0.52, 95% CI 0.42-0.65; CS: OR 0.44, 95% CI: 0.31-0.61), and breastfeeding (OR 0.50, 95% CI 0.36-0.69 among vaginal births). To the authors’ knowledge, there are no studies conducted in countries with health care systems comparable to the Italian one that addressed this aspect.

Most cases (63.2%) occurred in the northern Italian Regions, the first European SARS-CoV-2 spot [27] and one of the most affected by the virus. Consistently to the spread of the virus in the country, the contribution from central and southern Regions has been minimal before September 2020 – January 2021, the period characterised by the collapse of the adopted indicators. Even before the COVID-19 emergency, evidence had highlighted gaps in the quality of perinatal care in Italy and heterogeneous practices [13, 28]. In line with other studies [13, 28], central and southern areas showed poorer childbirth care outcomes compared to northern Regions, confirming the worse perinatal performances detected even before the pandemic, with higher rates of CS [18, 29], maternal [30] and neonatal [31] mortality, and a lower breastfeeding rate [32]. Therefore, it can be speculated that the increased virus circulation in the South contributed to the worsening of the reported indicators. Moreover, in the South, a slight improvement in some indicators such as labour companion, skin-to-skin, and no mother–child separation at birth was observed only starting from January-May 2022. This finding underscores that, despite the circulation of national interim guidance about childbirth care [33], not all the maternity units were ready to respond to the COVID emergency, probably because support for a positive childbirth experience through a high-quality, evidence-based, and respectful perinatal care is still not considered crucial everywhere. Despite the gradual improvement observed in all the indicators after the third phase of the wild-type virus, pre-pandemic levels were not reached in more than two years of health emergency. This highlights the insufficient preparedness of the Italian healthcare system.

Comparing vaginal births and CSs, significant differences for all the studied indicators stand out. Overall, 70.5% of newborns have not been separated by their mothers after vaginal birth *vs* 36.8% after CS, and the same was for skin-to-skin (66.2% *vs* 21.6%), rooming-in (81.1% *vs* 56.4%), and breastfeeding (88.0% *vs* 71.9%). Differences were also detected in the IMAgine EURO study for rooming-in (78.2% after vaginal birth *vs* 69.5% after CS) and breastfeeding within the first hour (70.3% *vs* 49.5%) [13], underlining the inequalities that still exist among different mode of delivery and perinatal care [34–36].

By focusing only on the Omicron period and vaccine uptake, among women considered protected by vaccine against moderate or severe COVID-19 disease, the presence of a companion during labour and childbirth, skin-to-skin contact and rooming-in were more frequent compared to unprotected. Vaccination has proven effective against moderate to severe COVID-19 [10, 37], and it may have reassured clinicians and encouraged them to protect the physiology of birth among vaccinated women.

Given the lack of studies that comprehensively investigate the quality of perinatal care, especially in high-income countries, and the wide range of definitions and indicators used to describe the labour companion support [13, 28], it is challenging to compare data from different countries or maternity units and follow them up over time. Despite the sound methodology and the large cohort size, the Italian study collided with the same issue Dowse and colleagues mentioned [12]: the lack of high-quality data on implementing family-centred care. In a pre-pandemic systematic review of 35 papers, mostly from high-income countries, it was identified a range from 1 to 98% in skin-to-skin practice. They also highlighted the challenge of comparing data across countries due to the heterogeneity of definitions. [38].

### Strengths and limitations

To the authors' knowledge, currently, this is one of the few papers assessing the quality of perinatal care provided to a large cohort of infected women who delivered in Italy during the SARS-CoV-2 emergency. It comprehensively analyses many multiple core indicators of childbirth care. Moreover, as shown by other papers [5, 13, 19], this is one of the few studies conducted in a high-income country. The 21-month data collection allowed to observe the trend of the virus circulation and its impact on the quality of care provided during different pandemic waves. However, it is important to note that no data were collected when the Delta variant, which was associated with a higher risk of severe maternal adverse outcomes [39], was predominant in Italy [40]. Another study limitation was that the indicators of interest lacked detailed definitions, i.e. duration of the presence of a labour companion, the phase during which the companion was present (labour, childbirth, after birth, or all of them), skin-to-skin duration, and type of breastfeeding (exclusive or mixed feeding, how to distinguish and to evaluate them). Due to the absence of a strict definition, an overestimation of the investigated good practices cannot be excluded. We also recorded a high percentage of cases with missing values; however, to control this possible bias, models were performed on multi-imputed data.

### Research proposal

By analysing data collected for the present study we recognised the lack of accurate and common definitions of perinatal care indicators across countries. The scoping review of Bohren and colleagues identified a wide range of definitions for labour companion [19]. We endorse their proposal of “Reporting companionship separately for labour, birth, and postnatal periods, or as a composite across this continuum to ensure that measurement reflects the complexities of implementation”. We also support the conclusion of Brimdyr and colleagues, who underline the need for a “universally recognised definition of the procedure of skin-to-skin contact after birth” [4]. In addition, we propose the establishment of a panel of experts who can collaborate to identify a minimum but comprehensive core set of childbirth care indicators and develop standardized definitions that can be easily adopted by researchers and programmers and adapted to different care settings. From the perspective of participatory science [41], this procedure should be validated by “representatives of parents”.

Once an agreement on the indicators and their definition is reached, it is crucial to periodically monitor and report the labour and childbirth care quality and a prospective data collection based on sound methodology and identification of self-report instruments measuring women's experiences of maternity care [42] can be the best way to reach this aim. Linking these results could provide more reliable information and deeper insights.

## Conclusions

Despite the primary outcome for all pregnant women being a positive childbirth experience fulfilling their expectations [12], as supported by the WHO guidelines on intrapartum care [1], the few published data relating to the quality of birth care during the pandemic highlight that this issue is still not central to global health policies. Collecting this data during the pandemic in Italy was challenging, especially in the initial phase of the emergency that unexpectedly hit the country. Nevertheless, the foresight to recognize their importance allowed to identify the principal critical issues of childbirth care. Despite good physical maternal and perinatal outcomes, the emotional support of a companion of choice and a psychologically safe environment during labour, birth and postpartum was lacking for too many women. The question remains: will we be able to treasure this lesson and value the importance of addressing these issues in the future?

### Supplementary Information

Additional file 1: Appendix. The ItOSS national network of maternity units. Table S1a. Perinatal care offered to SARS-CoV-2 positive women who had a vaginal birth during the COVID-19 pandemic in Italy. Table S1b. Perinatal care offered to SARS-CoV-2 positive women who underwent caesarean section during the COVID-19 pandemic in Italy. Table S2a. Mutually adjusted odds ratios for the reported variables among women who had a vaginal birth. Logistic regression models on imputed data. Table S2b. Mutually adjusted odds ratios for the reported variables among women who underwent caesarean section. Logistic regression models on imputed data. Table S3a. Mutually adjusted odds ratios for the reported variables among women who had a vaginal birth. Logistic regression models on complete cases. Table S3b. Mutually adjusted odds ratios for the reported variables among women who underwent caesarean section. Logistic regression models on complete cases.

## Data Availability

The data presented in this study are available on request from the corresponding author.

## Abbreviations

CI: Confidence interval
COVID-19: Coronavirus disease of 2019
CS: Caesarean section
INIH: Italian National Institute of Health
OR: Odds ratio
SARS-CoV-2: Severe acute respiratory syndrome coronavirus 2
WHO: World Health Organization

## References

[1] World Health Organization. WHO recommendations: intrapartum care for a positive childbirth experience. Geneva: World Health Organization. ; 2018. https://apps.who.int/iris/bitstream/handle/10665/260178/9789241550215-eng.pdf;sequence=1. Accessed 23 June 2023. 30070803

[2] Moore ER, Anderson GC, Bergman N, Dowswell T (2012). Early skin-to-skin contact for mothers and their healthy newborn infants. Cochrane Database Syst Rev.

[3] Bohren MA, Hofmeyr GJ, Sakala C, Fukuzawa RK, Cuthbert A (2017). Continuous support for women during childbirth. Cochrane Database Syst Rev.

[4] Brimdyr K, Stevens J, Svensson K, Blair A, Turner-Maffei C, Grady J (2023). Skin-to-skin contact after birth: developing a research and practice guideline. Acta Paediatr.

[5] Adesanya AM, Barrett S, Moffat M, Aquino MRJ, Nicholson W, Turner G (2022). Impact of the COVID-19 pandemic on expectant and new parents’ experience of pregnancy, childbirth, breast feeding, parental responsiveness and sensitivity, and bonding and attunement in high-income countries: a systematic review of the evidence. BMJ Open.

[6] Wesołowska A, Orczyk-Pawiłowicz M, Bzikowska-Jura A, Gawrońska M, Walczak B (2022). Protecting breastfeeding during the COVID-19 pandemic: a scoping review of perinatal care recommendations in the context of maternal and child well-being. Int J Environ Res Public Health.

[7] Lalor JG, Sheaf G, Mulligan A, Ohaja M, Clive A, Murphy-Tighe S (2023). Parental experiences with changes in maternity care during the Covid-19 pandemic: a mixed-studies systematic review. Women Birth.

[8] Corsi E, Maraschini A, Perrone E, Salvatore MA, D’aloja P, Donati S (2020). The preparedness of the Italian obstetric surveillance system in the response to the emergency of the SARS-CoV-2 pandemic: methodological aspects of a population-based study. Epidemiol Prev.

[9] Donati S, Corsi E, Maraschini A, Salvatore MA, ItOSS-COVID-19 Working Group (2022). SARS-CoV-2 Infection among hospitalised pregnant women and impact of different viral strains on COVID-19 severity in Italy: a national prospective population-based cohort study. BJOG.

[10] Corsi Decenti E, Salvatore MA, Mandolini D, Donati S, Italian Obstetric Surveillance System COVID-19 Working Group (2023). Vaccination against SARS-CoV-2 in pregnancy during the Omicron wave: the prospective cohort study of the Italian obstetric surveillance system. Clin Microbiol Infect.

[11] Donati S, Corsi E, Salvatore MA, Maraschini A, Bonassisa S, Casucci P (2021). Childbirth Care among SARS-CoV-2 positive women in Italy. Int J Environ Res Public Health.

[12] Dowse G, Perkins EJ, Stein HM, Chidini G, Danhaive O, Elsayed YN (2023). Born into an isolating world: family-centred care for babies born to mothers with COVID-19. EClinicalMedicine.

[13] Lazzerini M, Covi B, Mariani I, Drglin Z, Arendt M, Nedberg IH (2022). Quality of facility-based maternal and newborn care around the time of Childbirth during the COVID-19 pandemic: online survey investigating maternal perspectives in 12 countries of the WHO European Region. Lancet Reg Health Eur.

[14] White IR, Royston P, Wood AM (2011). Multiple imputation using chained equations: issues and guidance for practice. Stat Med.

[15] Rubin DB (1987). Multiple imputation for nonresponse in surveys.

[16] Vousden N, Bunch K, Morris E, Simpson N, Gale C, O’Brien P (2021). The incidence, characteristics and outcomes of pregnant women hospitalized with symptomatic and asymptomatic SARS-CoV-2 Infection in the UK from March to September 2020: a national cohort study using the UK Obstetric Surveillance System (UKOSS). PLoS ONE.

[17] Overtoom EM, Rosman AN, Zwart JJ, Vogelvang TE, Schaap TP, van den Akker T (2022). SARS-CoV-2 infection in pregnancy during the first wave of COVID-19 in the Netherlands: a prospective nationwide population-based cohort study (NethOSS). BJOG.

[18] Directorate-General for Digitisation, Health Information System and Statistics. Statistical Office. Certificate of childbirth assistance. Analysis of the birth event –Year 2019. Rome., 2021. https://www.salute.gov.it/imgs/C_17_pubblicazioni_3076_allegato.pdf. Accessed 23 June 2023.

[19] Bohren MA, Hazfiarini A, Vazquez Corona M, Colomar M, De Mucio B, Tunçalp Ö (2023). From global recommendations to (in)action: a scoping review of the coverage of companion of choice for women during labour and birth. PLOS Glob Public Health.

[20] Bohren MA, Vogel JP, Hunter EC, Lutsiv O, Makh SK, Souza JP (2015). The mistreatment of women during childbirth in health facilities globally: a mixed-methods systematic review. PLoS Med.

[21] Directorate-General for Digitisation, Health Information System and Statistics. Statistical Office. Certificate of childbirth assistance. Analysis of the birth event –Year 2021. Rome., 2021. https://www.salute.gov.it/imgs/C_17_pubblicazioni_3264_allegato.pdf. Accessed 23 June 2023.

[22] Lauria L, Lamberti A, Buoncristiano M, Bonciani M, Andreozzi S. Pre-and post-natal assistance: promotion and assessment of operational models quality. The 2008–2009 and 2010–2011 surveys (in Italian). Rome: Italian National Institutes of Health; 2012. (ISTISAN repots 12/39). https://www.dors.it/documentazione/testo/201301/12_39_web.pdf. Accessed 23 June 2023.

[23] Dennis CL, Shiri R, Brown HK, Santos HP, Schmied V, Falah-Hassani K (2019). Breastfeeding rates in immigrant and non-immigrant women: a systematic review and meta-analysis. Matern Child Nutr.

[24] Socio-demographic and environmental statistics directorate Istat – National Institute of Statistics. Pregnancy, childbirth and breastfeeding in Italy – year 2013. Rome., 2014. https://www.istat.it/it/files//2014/12/Pregnancy-childbirth-breastfeeding-2013.pdf. Accessed 23 June 2023.

[25] Cammu H, Martens G, Keirse MJ (2011). Mothers’ level of education and childbirth interventions: a population-based study in Flanders, Northern Belgium. Birth.

[26] Wako WG, Wayessa Z, Fikrie A (2022). Effects of maternal education on early initiation and exclusive breastfeeding practices in sub-saharan Africa: a secondary analysis of demographic and health surveys from 2015 to 2019. BMJ Open.

[27] Cerqua A, Di Stefano R (2022). When did coronavirus arrive in Europe?. Stat Methods Appt.

[28] Lazzerini M, Covi B, Mariani I, Giusti A, Pessa Valente E, IMAgiNE EURO Study Group (2022). Quality of care at Childbirth: findings of IMAgiNE EURO in Italy during the first year of the COVID-19 pandemic. Int J Gynaecol Obstet.

[29] Montilla P, Merzagora F, Scolaro E, Requejo J, Ricciardi W, Meli E (2020). Lessons from a multidisciplinary partnership involving women parliamentarians to address the overuse of caesarean section in Italy. BMJ Glob Health.

[30] Donati S, Maraschini A, Lega I, D’Aloja P, Buoncristiano M, Manno V, Regional Maternal Mortality Working Group (2018). Maternal mortality in Italy: results and perspectives of record-linkage analysis. Acta Obstet Gynecol Scand.

[31] Simeoni S, Frova L, De Curtis M (2019). Inequalities in infant mortality in Italy. Ital J Pediatr.

[32] Pizzi E, Salvatore MA, Donati S, Andreozzi S, Battilomo S, Privitera MG, National Institute of Health (INIH). Istituto Superiore di Sanità – Italian. The Surveillance System on Children aged 0–2: purpose, methodology and results of the 2018–2019 data collection. Rome, 2022. https://www.iss.it/documents/20126/6703853/Rapporto_finale_+2016_sorveglianza+bambini+0-2+anni.pdf/ce1491d3-8e43-e885-d0e3-8d45a6ef73f3?t=1669291538200. Accessed 23 June 2023.

[33] Giusti A, Zambri F, Marchetti F, Corsi E, Preziosi J, Sampaolo L (2021). COVID-19 and pregnancy, Childbirth, and breastfeeding: the interim guidance of the Italian National Institute of Health. Epidemiol Prev.

[34] Ali NB, Priyanka SS, Bhui BR, Herrera S, Azad MR, Karim A (2021). Prevalence and factors associated with skin-to-skin contact (SSC) practice: findings from a population-based cross-sectional survey in 10 selected districts of Bangladesh. BMC Pregnancy Childbirth.

[35] Wu HL, Lu DF, Tsay PK (2022). Rooming-in and breastfeeding duration in first-time mothers in a modern postpartum care center. Int J Environ Res Public Health.

[36] Regan J, Thompson A, DeFranco E (2013). The influence of mode of delivery on breastfeeding initiation in women with a prior cesarean delivery: a population-based study. Breastfeed Med.

[37] Watanabe A, Yasuhara J, Iwagami M, Miyamoto Y, Yamada Y, Suzuki Y (2022). Peripartum outcomes Associated with COVID-19 vaccination during pregnancy: a systematic review and meta-analysis. JAMA Pediatr.

[38] Abdulghani N, Edvardsson K, Amir LH (2018). Worldwide prevalence of mother-infant skin-to-skin contact after vaginal birth: a systematic review. PLoS ONE.

[39] Favre G, Maisonneuve E, Pomar L, Daire C, Poncelet C, Quibel T (2023). Maternal and perinatal outcomes following pre-delta, Delta, and Omicron SARS-CoV-2 variants Infection among unvaccinated pregnant women in France and Switzerland: a prospective cohort study using the COVI-PREG registry. Lancet Reg Health Eur.

[40] Istituto Superiore di Sanità – Italian National Institute of Health (INIH). Monitoraggio delle varianti del virus SARS-CoV-2 di interesse in sanità pubblica in Italia. ; 2023. https://www.epicentro.iss.it/coronavirus/sars-cov-2-monitoraggio-varianti-rapporti-periodici. Accessed 23 June 2023.

[41] Laureij LT, Depla AL, Kariman SS, Lamain-de Ruiter M, Ernst-Smelt HE, Hazelzet JA (2023). Women’s experiences with using patient-reported outcome and experience measures in routine perinatal care in the Netherlands: a mixed-methods study. BMJ Open.

[42] Beecher C, Greene R, O’Dwyer L, Ryan E, White M, Beattie M (2021). Measuring women’s experiences of maternity care: a systematic review of self-report survey instruments. Women Birth.

