# Supplementary material for: Perinatal care in SARS-CoV-2 infected women: the lesson learnt from a national prospective cohort study during the pandemic in Italy
Source: BMC Public Health. 2023 Dec 21;23:2562. doi: 10.1186/s12889-023-17390-0 (PMC10740257; doi:10.1186/s12889-023-17390-0)
Supplement: Supplementary file 1 — Additional file 1: Appendix. The ItOSS national network of maternity units. Table S1a. Perinatal care offered to SARS-CoV-2 positive women who had a vaginal birth during the COVID-19 pandemic in Italy. Table S1b. Perinatal care offered to SARS-CoV-2 positive women who underwent caesarean section during the COVID-19 pandemic in Italy. Table S2a. Mutually adjusted odds ratios for the reported variables among women who had a vaginal birth. Logistic regression models on imputed data. Table S2b. Mutually adjusted odds ratios for the reported variables among women who underwent caesarean section. Logistic regression models on imputed data. Table S3a. Mutually adjusted odds ratios for the reported variables among women who had a vaginal birth. Logistic regression models on complete cases. Table S3b. Mutually adjusted odds ratios for the reported variables among women who underwent caesarean section. Logistic regression models on complete cases. [file 12889_2023_17390_MOESM1_ESM.docx]

**Supplementary Information**

The online version contains supplementary material available at

**Appendix** The ItOSS national network of maternity units. **Table S1a** Perinatal care offered to SARS-CoV-2 positive women who had a vaginal birth during the COVID-19 pandemic in Italy. **Table S1b** Perinatal care offered to SARS-CoV-2 positive women who underwent caesarean section during the COVID-19 pandemic in Italy. **Table S2a** Mutually adjusted odds ratios for the reported variables among women who had a vaginal birth. Logistic regression models on imputed data. **Table S2b** Mutually adjusted odds ratios for the reported variables among women who underwent caesarean section. Logistic regression models on imputed data. **Table S3a** Mutually adjusted odds ratios for the reported variables among women who had a vaginal birth. Logistic regression models on complete cases. **Table S3b** Mutually adjusted odds ratios for the reported variables among women who underwent caesarean section. Logistic regression models on complete cases.

**Appendix** The ItOSS national network of maternity units.

# Piedmont Region

Elena Amoruso *Ospedale Sant'Andrea Vercelli;* Alberto Arnulfo, Enrico Finale *Stabilimento Ospedaliero Castelli Verbania;* Rossella Attini, Marisa Biasio, Luca Marozio, Clara Monzeglio OIRM *Sant’Anna -* *AOU Città della Salute e della Scienza di Torino;* Maria Bertolino, Andrea Guala *Ospedale San Biagio Domodossola;* Silvia Bonassisa, Alberto De Pedrini *Ospedale Maggiore della Carità Novara;* Mario Canesi, Sara Cantoira *Ospedale Maria Vittoria Torino;* Paola Capelli *Istituto SS. Trinità Borgomanero;* Ilaria Careri, *Ospedale Martini Torino;* Luigi Carratta *Ospedale S. Spirito Casale Monferrato;* Ilaria Costaggini *Ospedale degli Infermi Rivoli;* Tania Cunzolo *Presidio Osp. Cardinal G. MASSAIA Asti;* Enza De Fabiani, Andrea Villasco *Azienda Ospedaliera Ordine Mauriziano Torino;* Cinzia Diano *Ospedale Maggiore Chieri;* Fiorenza Droghini, Paola Rota *Ospedale Santa Croce Moncalieri;* Daniela Kozel, Vittorio Aguggia *Ospedale Civile SS. Antonio e Biagio Alessandria;*  Francesca Maraucci *Ospedale degli infermi Biella;* Gisella Martinotti *Ospedale SS. Pietro e Paolo Borgosesia;* Maria Milano, Antonia Novelli *Ospedale Civile Mondovì;* Giovanna Oggè *Ospedale maggiore SS. Annunziata Savigliano;* Simona Pelissetto *Ospedale Civile di Ivrea;* Pasqualina Russo *Presidio Osp. riunito Ciriè;* Manuela Scatà *Ospedale Michele e Pietro Ferrero di Verduno;* Federico Tuo, Valentina Casagrande *Ospedale San Giacomo Novi Ligure/Tortona;* Concetta Vardè *Ospedale Agnelli Pinerolo;* Elena Vasario *Azienda Ospedaliera S. Croce e Carle Cuneo*; Daniela Ventrella *Ospedale Civico Chivasso*

# Valle D’Aosta Region

Livio Leo *Ospedale* *Umberto Parini Aosta*

# Liguria Region

Silvia Andrietti *ASL1 Imperiese*; Federica Baldi *Ospedale San Paolo Savona*; Angelo Cagnacci, Federica Laraud *IRCCS AOU San Martino*; Franco Camandona, Domenico Grimaldi *Ospedale Galliera di Genova*; Maria Franca Corona, Massimiliano Leoni *Ospedale Civile Sant’Andrea La Spezia*; Paolo Massirio, Luca Ramenghi *IRCCS Giannina Gaslini*

# Lombardy Region

Debora Balestrieri *Ospedale di Cittiglio;* Federica Baltaro *Ospedale Niguarda di Milano;* Pietro Barbacini, Elisabetta Venegoni *Ospedale di Magenta;* Michele Barbato *Ospedale di Melegnano;* Lorena Barbetti *Ospedale di Esine;* Paolo Beretta *Ospedale di Como;* Bruno Bersellini *Ospedale di Sondrio;* Stefano Bianchi *Ospedale San Giuseppe di Milano;* Antonia Botrugno *Ospedale di Casalmaggiore;* Donatella Bresciani *Ospedale di Desenzano;* Alessandro Bulfoni *Pio X Humanitas di Milano;* Carlo Bulgheroni *Ospedale di Gallarate;* Orlando Caruso, Elena Pinton *Ospedale di Chiari;* Massimo Ciammella *Ospedale di Seriate;* Elena Crestani, Giulia Pellizzari *Ospedale di Pieve di Coriano;* Antonella Cromi *Ospedale di Varese;* Serena Dalzero, Nikita Alfieri *Ospedale San Paolo di Milano;* Rosa Di Lauro, Carla Foppoli *Ospedale di Sondalo;* Patrizia D'Oria, *Ospedale di Alzano;* Santina Ermito *Ospedale di Piario;* Massimo Ferdico *Ospedale di Vimercate;* Maria Fogliani, Guido Stevanazzi *Ospedale di Legnano-Cuggiono;* Roberto Fogliani *Ospedale di Sesto San Giovanni;* Ambrogio Frigerio *Ospedale di Rho;* Eleonora Fumagalli *Ospedale Macedonio Melloni ASST FBF-Sacco di Milano;* Roberto Garbelli *Brescia Istituto Clinico S. Anna;* Daniela Gatti *Ospedale di Manerbio;* Giampaolo Grisolia, Serena Varalta *Ospedale di Mantova;* Paolo Guarnerio *Ospedale San Carlo di Milano;* Enrico Iurlaro, Marta Tondo *IRCCS Cà Granda Ospedale Maggiore Policlinico-Mangiagalli Milano;* Stefano Landi *Ospedale di Gravedona;* Mario Leonardi *Ospedale di Iseo;* Stefania Livio, Chiara Tasca *Ospedale Buzzi ASST FBF-Sacco di Milano;* Anna Locatelli *Ospedale di Carate;* Giuseppe Losa *Ospedale di Melzo;* Massimo Lovotti *Como Valduce;* Anna Minelli *Ospedale di Gavardo;* Luisa Muggiasca *Ospedale di Garbagnate;* Giuseppe Nucera *Ospedale di Busto Arsizio;* Alessandra Ornati *Ospedale di Vigevano;* Luisa Patanè *ASST Papa Giovanni XXIII Bergamo;* Antonio Pellegrino *Ospedale di Lecco;* Francesca Perotti, Arsenio Spinillo *Fondazione IRCCS Policlinico San Matteo di Pavia;* Armando Pintucci *Ospedale di Desio;* Ezio Pozzi *Ospedale di Broni Stradella- Ospedale di Voghera,* Federico Prefumo *Spedali Civili di Brescia;* Anna Catalano *Brescia Fondazione Poliambulanza;* Aldo Riccardi *Ospedale di Cremona;* Alessia Chiesa *Ospedale di Ponte San Pietro;* Tazio Sacconi *Ospedale di Asola;* Valeria Savasi, Silvia Corti *Ospedale Sacco di Milano;* Ubaldo Seghezzi *Ospedale di Saronno;* Vincenzo Siliprandi *Ospedale di Crema;* Marco Soligo, Beatrice Negri *Ospedale di Lodi;* Paolo Valsecchi *Ospedale San Raffaele;* Laura Vassena *Ospedale di Merate;* Federica Brunetti, Patrizia Vergani *Fondazione MBBM Ospedale San Gerardo Monza;* Antonella Villa *Ospedale di Treviglio;* Matteo Zanfrà *Ospedale di Tradate;* Alberto Zanini *Ospedale di Erba*

# Autonomous province of Bozen

Martin Steinkasser, Micaela Veneziano *Ospedale Centrale di Bolzano*

# Autonomous province of Trento

Pietro Dal Rì, Fabrizio Taddei *UO Rovereto*; Roberto Luzietti *UO Cles*; Saverio Tateo *UO Trento*; Fabrizia Tenaglia *UO Cavalese*

# Veneto Region

Giuseppe Angeloni *Ospedale di Piove di Sacco;* Antonio Azzena *Ospedale di Vittorio Veneto;* Gianluca Babbo *Ospedale di Portogruaro*; Roberto Baccichet, Cristina Napolitano *Ospedale di Oderzo;* Valentino Bergamini *Ospedale Borgo Trento;* Luca Bergamini *Ospedale di Chioggia;* Enrico Busato, Monica Zannol *Ospedale di Treviso;* Pietro Catapano, Marco Gentile *Ospedale Mater Salutis – Legnago;* Marcello Ceccaroni *Ospedale Sacro Cuore don Calabria Negrar;* Gianluca Cerri *Ospedale SS. Giovanni e Paolo – Venezia;* Andrea Cocco *Ospedale di Asiago;* Carlo Dorizzi *Ospedale di Schiavonia;* Laura Favretti *Ospedale S. Maria del Prato – Feltre;* Riccardo Federle, Antonino Lo Re *Ospedale P. Pederzoli - Casa di cura Privata Spa;* Massimo Franchi, Marina Sangaletti *Azienda Ospedaliera di Verona;* Franco Garbin *Ospedale di Dolo;* Maria Teresa Gervasi, Daniela Truscia *Azienda Ospedaliera di Padova;* Dimosthenis Kaloudis *Ospedale di San Bonifacio;* Domenico Lagamba *Ospedale di Castelfranco Veneto;* Giovanni Martini *Ospedale di Valdagno;* Carlo Maurizio *Ospedale di Mirano;* Yoram J. Meir *Ospedale di Bassano del Grappa;* Alessia Pozzato *Ospedale di Adria* - *Ospedale di Rovigo;* Marcello Rigano *Ospedale di Camposampiero;* Cesare Romagnolo *Ospedale all’Angelo di Mestre;* Roberto Rulli *Ospedale di Cittadella;* Giuseppe Sacco *Ospedale di San Donà di Piave;* Maria Grazia Salmeri *Ospedale di Montebelluna;* Marcello Scollo *Ospedale di Santorso;* Francesco Sinatra *Ospedale di Conegliano;* Gianluca Straface *Casa di cura Abano;* Fabio Gianpaolo Tandurella *Ospedale di Pieve di Cadore* e *Ospedale San Martino – Belluno;* Marco Torrazzina *Ospedale di Bussolengo* - *Ospedale di Villafranca;* Paolo Lucio Tumaini *Ospedale di Arzignano;* Giuliano Zanni *Ospedale di Vicenza*

# Friuli-Venezia Giulia Region

Emanuele Ancona Ospedale *S. Giorgio di Pordenone;* Michela De Agostini *Ospedale di Palmanova;* Gianpaolo Maso, Alice Sorz *IRCSS Burlo Garofolo Trieste;* Edlira Muharremi *S. M. degli Angeli Ospedale di Pordenone;* Alessandra Nicoletti *Ospedale S.Daniele di Tolmezzo;* Roberta Pinzano *Ospedale S.Maria dei Battuti-San Vito al Tagliamento;* Alessia Sala O*spedale Santa Maria della Misericordia-Udine;* Lucia Zanazzo, *Ospedale di Monfalcone*

# Emilia-Romagna Region

Lorenzo Aguzzoli, Alice Ferretti *Ospedale S.M. Nuova Reggio Emilia;* Patrizio Antonazzo, Lucrezia Pignatti *Ospedale Bufalini Cesena;* Angela Bandini, Isabella Strada *Ospedale G.B .Morgagni -L. Pierantoni Forlì;* Chiara Belosi *Ospedale degli Infermi Faenza;* Renza Bonini, Maria Cristina Ottoboni *Ospedale Guglielmo Da Saliceto Piacenza;* Fabrizio Corazza, Paola Pennacchioni *Ospedale Ss. Annunziata Cento;* Fabio Facchinetti, Giliana Ternelli *Azienda Ospedaliero-Universitaria Modena*; Alessandro Ferrari*,* Cristina Pizzi, *Ospedale S.M. Bianca Mirandola;* Tullio Ghi, Stefania Fieni *Azienda Ospedaliero-Universitaria Parma;* Maria Cristina Galassi, Federica Richieri, *Nuovo Ospedale Civile Di Sassuolo S.P.A.;* Giuseppe Comerci, Carlotta Matteucci *Ospedale S.M. Delle Croci Ravenna;* Pantaleo Greco, Danila Morano *Azienda Ospedaliero-Universitaria Ferrara;* Marinella Lenzi, Ilaria Cataneo, *Ospedale Maggiore Bologna;* Gialuigi Pilu, Marisa Bisulli, *Azienda Ospedaliero-Universitaria Bologna;* Maria Cristina Selleri *Ospedale di Bentivoglio;* Federico Spelzini, Lidia Di Cerbo, *Ospedale Infermi Rimini;* Giulia Pellizzari*,* Francesca Tassinati *Ospedale B. Ramazzini Carpi;* Stefano Zucchini, Barbara Paccaloni, *Ospedale S.M. della Scaletta Imola*

# Tuscany Region

Andrea Antonelli, Carlotta Boni *Ospedale Civile Cecina;* Maria Paola Belluomini, *S. Francesco Barga - PO Valle del Serchio* e *Generale Provinciale Lucca - PO San Luca,* Rosalia Bonura, *S. Maria della Gruccia - Ospedale del Valdarno,* Stefano Braccini, *SS. Cosimo e Damiano Pescia - Osp della Valdinievole,* Giacomo Bruscoli e Pasquale Mario Florio, *Nuovo Ospedale San Jacopo di Pistoia,* Giovanna Casilla, *SS. Giacomo e Cristoforo Massa - PO Zona Apuana,* Anna Franca Cavaliere, *Ospedale Santo Stefano Prato,* Marco Cencini, *Ospedali Riuniti della Val di Chiana,* Venere Coppola e Laura Migliavacca, *Ospedale Misericordia Grosseto,* Barbara De Santi, *PO Felice Lotti Pontedera,* Paola Del Carlo, *Ospedale S.Giovanni Di Dio Torregalli,* Carlo Dettori, *Nuovo Ospedale di Borgo S.Lorenzo,* Mariarosaria Di Tommaso e Serena Simeone, *Careggi - CTO Firenze - AOU,* Giuseppe Eremita, *Civile Elbano Portoferraio,* Sara Failli, *Ospedale Area Aretina Nord Arezzo,* Paolo Gacci, *S.M. Annunziata Bagno a Ripoli - Osp Fiorentino Sud Est,* Alessandra Meucci, *Le Scotte Siena - Azienda ospedaliera universitaria,* Filippo Ninni, *Riuniti Livorno,* Barbara Quirici, *Ospedale Unico Versilia,* Alessia Sacchi, *Ospedale dell'Alta Val d'Elsa Poggibonsi,* Cristina Salvestroni, *Ospedale S. Giuseppe Empoli,* Sara Zullino, *Ospedali Pisani Pisa - Az universitaria*

# Umbria Region

Nazzareno Cruciani, Fabrizio Damiani *Ospedale San Giovanni Battista Foligno*; Leonardo Borrello *Azienda Ospedaliera Santa Maria di Terni*; Gian Carlo Di Renzo, Giorgio Epicoco *Azienda Ospedaliera Santa Maria della Misericordia di Perugia*; Ugo Indraccolo, Donatello Torrioli, *Ospedale di Città di Castello*

# Marche Region

Andrea Ciavattini, Sara D’Eusanio *AOU - Ospedali Riuniti di Ancona*; Filiberto Di Prospero *Ospedale di Civitanova Marche*; Rebecca Micheletti, Claudio Cicoli *Azienda Ospedaliera Ospedali Riuniti Marche Nord*

# Lazio Region

Francesco Antonino Battaglia, Immacolata Marcucci *PO Santa Maria Goretti Latina;* Leonardo Boccuzzi, Patrizia Ruocco *Ospedale De Santis di Genzano*; Marco Bonito *Ospedale San Pietro Fatebenefratelli Roma;* Maria Clara D’Alessio *San Filippo Neri Roma*; Carlo De Angelis *Casa di Cura Fabia Mater Roma*; Donatella Dell’Anna *Ospedale S. Eugenio*; Daniele Di Mascio, Paola Pecilli *Umberto I - Policlinico di Roma;* Sascia Moresi, Sergio Ferrazzani, Silvia Salvi *Policlinico Universitario Fondazione Agostino Gemelli - Roma;* Gregorio Marco Galati *Ospedale Madre Giuseppina Vannini Istituto delle Figlie di S. Camillo Roma*; Maria Grazia Frigo *Fatebenefratelli San Giovanni Calibita - Isola Tiberina;* Paolo Gastaldi *Ospedale Santo Spirito Roma*; Rita Gentile *Presidio Ospedaliero Giovan Battista Grassi Ostia;* Giovanni Grossi *Ospedale Sandro Pertini Roma*; Giorgio Nicolanti, Patrizio Raggi *Ospedale Belcolle Viterbo;* Flavia Pierucci *Azienda Ospedaliera San Camillo Forlanini Roma*; Giancarlo Paradisi, Maria Rita Pecci *Ospedale Fabrizio Spaziani Frosinone*; Giovanni Testa *Casa di cura Città di Aprilia*; Barbara Vasapollo *Policlinico Casilino Roma;* Barbara Villaccio *Ospedale San Pietro Fatebenefratelli Roma*

# Abruzzo Region

Fabio Benucci *Sant’Omero;* Paola Caputo *Sulmona;* Sandra Di Fabio, Maurizio Guido *L’Aquila;* Antonio Di Francesco *Lanciano;* Francesca di Sebastiano, Diego Gazzolo, Marco Liberati *Chieti;* Anna Marcozzi *Teramo;* Francesco Matrullo *Vasto;* Maurizio Rosati, Gabriella Scorpio *Pescara;* Giuseppe Ruggeri *Avezzano*; Alessandro Santarelli *Ospedale di Sant’Omero*

# Molise Region

Daniela Simeone *Ospedale di Campobasso*

# Campania Region

Annalisa Agangi *Ospedale Evangelico Villa Betania*; Salvatore Ercolano *P.O. "S. Leonardo" di Castellammare di Stabia*, Luigi Cobellis, Annunziata Mastrogiacomo *Ospedale di Caserta*; Maria Vittoria Locci *AOU Federico II Napoli*

# Puglia Region

Luca Loiudice *Bari - Presidio Mater Dei;* Antonio Belpiede *Barletta 'Mons. Dimiccoli';* Mariano Cantatore *L. Bonomo Andria;* Ettore Cicinelli, Antonella Vimercati *Bari - Policlinico Ginecologia;* Aldo D’Aloia, Sabina Di Biase, Antonio Lacerenza *AOU “OO RR Foggia”;* Alessandro Dalfiero *Cerignola;* Gerardo D'Ambrogio *Galatina "Santa Caterina Novella";* Nicola Del Gaudio *Castellaneta;* Paolo Demarzo *San Severo Teresa Masselli Mascia;* Giovanni Di Vagno *Bari - San Paolo;* Giuseppe Laurelli *Casa Sollievo Dalla Sofferenza - S. Giovanni Rotondo;* Roberto Lupo *Gallipoli;* Nicola Macario *Altamura;* Antonio Malvasi, *Bari - Casa Di Cura Santa Maria;* Guido Maurizio, Elisabetta Monteduro, *Acquaviva "Miulli";* Andrea Morciano *Cardinale G. Panico Di Tricase;* Lucio Nichilo *Umberto I Corato;* Anna Maria Nimis *Francavilla Fontana;* Antonio Perrone *Lecce Vito Fazzi;* Elena Rosa Potì *Brindisi "Perrino";* Sabino Santamato *Monopoli Putignano;* Emilio Stola *Taranto;* Antonio Tau *Scorrano;* Mario Vicino *Bari - Di Venere;* Martino Vinci *Martina Franca*

# Basilicata Region

Giampiero Adornato *Policoro*; Francesco Bernasconi *Melfi*; Alfonso Chiacchio *Lagonegro*; Sergio Schettini, Rocco Paradiso *Azienda Ospedaliera Regionale San Carlo – Potenza*; Giuseppe Trojano *Matera*

# Calabria Region

Carmelina Ermio *Ospedale Jazzolino - Vibo Valentia*; Michele Morelli, Rossella Marzullo *Ospedale Annunziata – AO Cosenza;* Stefano Palomba *Ospedali Riuniti di Reggio Calabria;* Morena Rocca *Azienda Ospedaliera "Pugliese Ciaccio" di Catanzaro*

# Sicily Region

Vincenzo Aidala, *Castiglione Prestianni-Bronte;* Luigi Alio, Giuseppina Orlando *ARNAS Civico di Cristina Benfratelli-Palermo;* Maria Grazia Arena, Santo Recupero *S. Marco (ex V. Emanuele S. Bambino)-Catania* e *Osp. Generale-Lentini;* Salvatore Bevilacqua, Fabrizio Quartararo *Casa di cura Candela SPA-Palermo;* Rocco Billone *Civico Partinico* e *Dei Bianchi-Corleone;* Giuseppe Bonanno, *Maria Paternò Arezzo-Ragusa;* Antonio Bucolo, *Umberto I-Siracusa;* Claudio Campione, *Casa di cura prof. Falcidia-Catania;* Giuseppe Canzone, *S. Cimino-Termini Imerese;* Angelo Caradonna, *V. Emanuele II-Castelvetrano;* Sebastiano Caudullo e Cosimo Raffone, *AO Papardo-Messina;* Giovanni Cavallo, *PO Maggiore-Modica;* Antonio Cianci, Michele Fichera *V. Emanuele Rodolico-Catania;* Salvatore Corsello, Sergio Di Salvo *Casa di cura Villa Serena-Palermo;* Gaspare Cucinella, Maria Elena Mugavero *Villa Sofia - Cervello-Palermo;* Rosario D'Anna, *AOU G. Martino-Taormina,* Maria Rosa D'anna, *Buccheri La Ferla-Palermo;* Maria Di Costa, *Basilotta-Catania;* Giuseppe Ettore, *ARNAS Garibaldi Nesima-Catania;* Giovanni Falzone, Marta Fauzia *Umberto I-Nicosia;* Roberto Fazio, *G. Fogliani-Milazzo* e *Lipari;* Matteo Giardina *Ospedale Paolo Borsellino di Marsala*; Michele Gulizzi e Francesco La Mantia, *G. F. Ingrassia-Palermo;* Laura Giambanco, *S. Antonio Abate-Erice* e *B. Nagar-Pantelleria;* Salvatore Incandela, *S. Giovanni di Dio -Agrigento* e *Giovanni Paolo II-Sciacca;* Lilli Maria Klein *S. Vincenzo-Enna* e *Barone Romeo-Patti;* Michele La Greca, Venera Mille, *M. SS. Addolorata-Biancavilla;* Luigi Li Calsi *S. Giacomo d'Altopasso-Licata;* Emilio Lo Meo, Paolo Scrollo *Cannizzaro-Catania;* Vincenzo Miceli, *S. Raffaele Giglio-Cefalù;* Maria Pia Militello, *S. Marta e S. Venera-Acireale;* Alfio Mirenna, *Istituto clinico Vidimura (ex Casa di cura Gretter e Lucina)-Catania;* Pietro Musso, *Abele Ajello-Mazara del Vallo;* Michele Palmieri, *V. Emanuele-Gela;* Concetta Remigia Pettinato, Angelo Tarascio *Gravina - Caltagirone-Caltagirone;* Vincenzo Scattarreggia *Barone Lombardo-Canicattì;* Antonio Schifano *R. Guzzardi-Vittoria;* Calogero Selvaggio *S. Elia-Caltanissetta;* Luigi Triolo *Casa di cure Triolo Zancla SPA-Palermo;* Renato Venezia *P. Giaccone-Palermo*

# Sardinia Region

Speranza Piredda *Civile Alghero;* Giangavino Peppi *Giovanni Paolo II - Olbia;* Giovanna Pittorra *S. Fracesco - Nuoro;* Gianfranco Depau *Nostra Signora della Mercede - Lanusei;* Gianfranco Puggioni *S. Martino - Oristano;* Loredana Pagliara *Nostra Signora di Bonaria - San Gavino;* Giulietta Ibba *CTO - Iglesias;* Caterina Tronci, *SS Trinità - Cagliari;* Giampiero Capobianco, *AOU Sassari;* Alessandra Meloni *Duilio Casula Monserrato AOU - Cagliari;* Francesca Palla *S. Michele AO Brotzu – Cagliari*

**Table S1a** Perinatal care offered to SARS-CoV-2 positive women who had a vaginal birth during the COVID-19 pandemic in Italy.

|  | **Wild-type virus, phase 1**  **(25 February - May 31, 2020)** | | **Wild-type virus, phase 2**  **(June 1 - August 31, 2020)** | | **Wild-type virus, phase 3**  **(September 1, 2020 – January 31, 2021)** | | **Alpha variant**  **(February 1 - June 30, 2021)** | | **Omicron variant**  **(January 1 - May 31, 2022)** | | **Total** | |
| --- | --- | --- | --- | --- | --- | --- | --- | --- | --- | --- | --- | --- |
|  | **n** | **%** | **n** | **%** | **n** | **%** | **n** | **%** | **n** | **%** | **N** | **%** |
| Presence of labour companion |  |  |  |  |  |  |  |  |  |  |  |  |
| No | 110 | 54.7 | 37 | 48.1 | 773 | 70.6 | 261 | 66.6 | 865 | 57.3 | 2,046 | 62.5 |
| Yes | 91 | 45.3 | 40 | 51.9 | 322 | 29.4 | 131 | 33.4 | 645 | 42.7 | 1,229 | 37.5 |
| *missing* | *1* | *0.5* | *1* | *1.3* | *80* | *6.8* | *30* | *7.1* | *187* | *11.0* | *299* | *8.4* |
| Skin-to-skin |  |  |  |  |  |  |  |  |  |  |  |  |
| No | 133 | 71.9 | 31 | 43.7 | 463 | 44.6 | 112 | 29.1 | 334 | 22.3 | 1,073 | 33.8 |
| Yes | 52 | 28.1 | 40 | 56.3 | 576 | 55.4 | 273 | 70.9 | 1,165 | 77.7 | 2,106 | 66.2 |
| *missing* | *14* | *7.0* | *7* | *9.0* | *127* | *10.9* | *37* | *8.8* | *192* | *11.4* | *377* | *10.6* |
| Mother-child separation at birth |  |  |  |  |  |  |  |  |  |  |  |  |
| No | 125 | 65.8 | 62 | 81.6 | 680 | 64.7 | 271 | 70.9 | 1,086 | 74.5 | 2,224 | 70.5 |
| Yes | 65 | 34.2 | 14 | 18.4 | 371 | 35.3 | 111 | 29.1 | 371 | 25.5 | 932 | 29.5 |
| *missing* | *9* | *4.5* | *2* | *2.6* | *115* | *9.9* | *40* | *9.5* | *234* | *13.8* | *400* | *11.2* |
| Rooming-in |  |  |  |  |  |  |  |  |  |  |  |  |
| No | 39 | 20.0 | 8 | 10.4 | 252 | 23.1 | 73 | 18.5 | 242 | 16.2 | 614 | 18.9 |
| Yes | 156 | 80.0 | 69 | 89.6 | 840 | 76.9 | 322 | 81.5 | 1,248 | 83.8 | 2,635 | 81.1 |
| *missing* | *4* | *2.0* | *1* | *1.3* | *74* | *6.3* | *27* | *6.4* | *201* | *11.9* | *307* | *8.6* |
| Breastfeeding |  |  |  |  |  |  |  |  |  |  |  |  |
| No | 24 | 13.0 | 5 | 6.8 | 165 | 15.9 | 44 | 11.5 | 139 | 9.5 | 377 | 12.0 |
| At breast | 148 | 80.0 | 64 | 86.5 | 797 | 76.6 | 306 | 79.9 | 1,236 | 84.8 | 2,551 | 81.2 |
| Squeezed maternal milk | 13 | 7.0 | 5 | 6.8 | 79 | 7.6 | 33 | 8.6 | 82 | 5.6 | 212 | 6.8 |
| *missing* | *14* | *7.0* | *4* | *5.1* | *125* | *10.7* | *39* | *9.2* | *234* | *13.8* | *416* | *11.7* |

Percentages were calculated based on cases with known information.

The percentage of presence of labour companion was calculated among women who had a vaginal birth (n=3,574). For the other indicators, percentages were calculated among all livebirths by vaginal delivery (n=3,556).

**Table S1b** Perinatal care offered to SARS-CoV-2 positive women who underwent caesarean section during the COVID-19 pandemic in Italy.

|  | **Wild-type virus, phase 1**  **(25 February - May 31, 2020)** | | **Wild-type virus, phase 2**  **(June 1 - August 31, 2020)** | | **Wild-type virus, phase 3**  **(September 1, 2020 – January 31, 2021)** | | **Alpha variant**  **(February 1 - June 30, 2021)** | | **Omicron variant**  **(January 1 - May 31, 2022)** | | **Total** | |
| --- | --- | --- | --- | --- | --- | --- | --- | --- | --- | --- | --- | --- |
|  | **n** | **%** | **n** | **%** | **n** | **%** | **n** | **%** | **n** | **%** | **N** | **%** |
| Skin-to-skin |  |  |  |  |  |  |  |  |  |  |  |  |
| No | 93 | 95.9 | 35 | 83.3 | 475 | 84.1 | 179 | 84.0 | 489 | 69.5 | 1,271 | 78.4 |
| Yes | 4 | 4.1 | 7 | 16.7 | 90 | 15.9 | 34 | 16.0 | 215 | 30.5 | 350 | 21.6 |
| *missing* | *10* | *9.3* | *0* | *0.0* | *68* | *10.7* | *20* | *8.6* | *141* | *16.7* | *239* | *12.8* |
| Mother-child separation at birth |  |  |  |  |  |  |  |  |  |  |  |  |
| No | 24 | 23.5 | 14 | 36.8 | 184 | 32.7 | 72 | 32.7 | 306 | 43.3 | 600 | 36.8 |
| Yes | 78 | 76.5 | 24 | 63.2 | 378 | 67.3 | 148 | 67.3 | 401 | 56.7 | 1,029 | 63.2 |
| *missing* | *5* | *4.7* | *4* | *9.5* | *71* | *11.2* | *13* | *5.6* | *138* | *16.3* | *231* | *12.4* |
| Rooming-in |  |  |  |  |  |  |  |  |  |  |  |  |
| No | 50 | 51.0 | 12 | 31.6 | 285 | 49.1 | 114 | 53.5 | 251 | 35.7 | 712 | 43.6 |
| Yes | 48 | 49.0 | 26 | 68.4 | 295 | 50.9 | 99 | 46.5 | 453 | 64.3 | 921 | 56.4 |
| *missing* | *9* | *8.4* | *4* | *9.5* | *53* | *8.4* | *20* | *8.6* | *141* | *16.7* | *227* | *12.2* |
| Breastfeeding |  |  |  |  |  |  |  |  |  |  |  |  |
| No | 37 | 38.1 | 6 | 16.7 | 202 | 36.9 | 62 | 30.2 | 132 | 19.6 | 439 | 28.1 |
| At breast | 43 | 44.3 | 26 | 72.2 | 266 | 48.5 | 89 | 43.4 | 450 | 66.8 | 874 | 56.0 |
| Squeezed maternal milk | 17 | 17.5 | 4 | 11.1 | 80 | 14.6 | 54 | 26.3 | 92 | 13.6 | 247 | 15.8 |
| *missing* | *10* | *9.3* | *6* | *14.3* | *85* | *13.4* | *28* | *12.0* | *171* | *20.2* | *300* | *16.1* |

Percentages were calculated based on cases with known information.

Percentages were calculated among all livebirths by caesarean section (n=1,860).

**Table S2a** Mutually adjusted odds ratios for the reported variables among women who had a vaginal birth. Logistic regression models on imputed data.

|  | **Presence of a labour companion (Yes *vs* No)** | **Skin-to-skin**  **(Yes *vs* No)** | **No mother-child separation at birth (Yes *vs* No)** | | **Rooming-in (Yes *vs* No)** | | **Breastfeeding**  **(Yes *vs* No)** |
| --- | --- | --- | --- | --- | --- | --- | --- |
|  | **OR (95% CI)** | **OR (95% CI)** | **OR (95% CI)** | | **OR (95% CI)** | | **OR (95% CI)** |
| Pandemic phase |  |  |  | |  | | |
| Wild-type virus, phase 1  (25 February - May 31, 2020) | 1 | 1 | 1 | | 1 | | 1 |
| Wild-type virus, phase 2  (June 1 - August 31, 2020) | 1.33 (0.78-2.27) | 3.09 (1.71-5.58) | 1.69 (0.86-3.32) | | 1.36 (0.57-3.29) | | 1.87 (0.65-5.43) |
| Wild-type virus, phase 3  (September 1, 2020 – January 31, 2021) | 0.62 (0.46-0.86) | 4.31 (3.00-6.18) | 1.17 (0.81-1.67) | | 1.40 (0.89-2.20) | | 1.42 (0.86-2.35) |
| Alpha variant  (February 1 - June 30, 2021) | 0.75 (0.53-1.07) | 8.71 (5.77-13.16) | 1.71 (1.14-2.56) | | 2.07 (1.23-3.50) | | 2.07 (1.17-3.66) |
| Omicron variant  (January 1 - May 31, 2022) | 1.17 (0.87-1.59) | 13.54 (9.37-19.56) | 2.17 (1.51-3.12) | | 2.86 (1.79-4.56) | | 2.56 (1.52-4.31) |
| Age |  |  |  |  | |  |  |
| <30 years |  |  |  | |  | |  |
| 30-34 years |  |  |  | |  | |  |
| ≥35 years |  |  |  | |  | |  |
| Citizenship |  |  |  |  | |  |  |
| Not Italian | 1 |  |  | |  | | 1 |
| Italian | 1.47 (1.23-1.74) |  |  | |  | | 0.63 (0.45-0.88) |
| Level of education |  |  |  |  | |  |  |
| Low |  | 0.72 (0.56-0.92) |  | |  | | 0.56 (0.39-0.81) |
| Medium |  | 0.76 (0.59-0.97) |  | |  | | 0.79 (0.57-1.10) |
| High |  | 1 |  | |  | | 1 |
| Parity |  |  |  |  | |  |  |
| Nulliparous |  | 0.81 (0.69-0.97) | 0.80 (0.67-0.95) | |  | |  |
| Multiparous |  | 1 | 1 | |  | |  |
| Gestational age at birth |  |  |  |  | |  |  |
| <37 weeks |  | 0.32 (0.22-0.46) | 0.24 (0.17-0.33) | | 0.14 (0.09-0.20) | | 0.38 (0.23-0.62) |
| ≥37 weeks |  | 1 | 1 | | 1 | | 1 |
| COVID-19 pneumonia |  |  |  |  | |  |  |
| No |  | 1 | 1 | | 1 | |  |
| Yes |  | 0.55 (0.34-0.88) | 0.31 (0.20-0.48) | | 0.40 (0.24-0.68) | |  |
| Volume of deliveries of maternity units |  |  |  |  | |  |  |
| <1,000 | 1 | 1 |  | | 1 | | 1 |
| 1,000-1,999 | 1.17 (0.97-1.42) | 0.82 (0.66-1.03) |  | | 1.25 (0.95-1.66) | | 1.70 (1.23-2.36) |
| ≥2,000 | 0.81 (0.67-0.98) | 0.52 (0.42-0.65) |  | | 0.70 (0.53-0.93) | | 0.50 (0.36-0.69) |
| Geographical location of maternity units |  |  |  |  | |  |  |
| North Italy | 1 | 1 | 1 | | 1 | | 1 |
| Centre Italy | 0.39 (0.31-0.49) | 0.34 (0.27-0.44) | 0.40 (0.31-0.50) | | 0.16 (0.12-0.21) | | 0.31 (0.21-0.44) |
| South Italy | 0.18 (0.14-0.23) | 0.11 (0.08-0.13) | 0.11 (0.09-0.13) | | 0.03 (0.02-0.04) | | 0.06 (0.04-0.08) |

In the models performed by stepwise procedure, the following variables were considered: pandemic phase, age, citizenship, level of education, parity, gestational age at birth, COVID-19 pneumonia, volume of deliveries of maternity units, geographical location of maternity units.

**Table S2b** Mutually adjusted odds ratios for the reported variables among women who underwent caesarean section. Logistic regression models on imputed data.

|  | **Skin-to-skin**  **(Yes *vs* No)** | **No mother-child separation at birth (Yes *vs* No)** | | **Rooming-in (Yes *vs* No)** | **Breastfeeding**  **(Yes *vs* No)** |
| --- | --- | --- | --- | --- | --- |
|  | **OR (95% CI)** | **OR (95% CI)** | | **OR (95% CI)** | **OR (95% CI)** |
| Pandemic phase |  |  | |  | |
| Wild-type virus, phase 1  (25 February - May 31, 2020) | 1 | 1 | | 1 | 1 |
| Wild-type virus, phase 2  (June 1 - August 31, 2020) | 3.08 (0.80-11.84) | 1.16 (0.48-2.80) | | 1.50 (0.55-4.11) | 1.68 (0.59-4.78) |
| Wild-type virus, phase 3  (September 1, 2020 – January 31, 2021) | 4.82 (1.71-13.60) | 1.73 (1.00-2.99) | | 1.30 (0.73-2.33) | 1.25 (0.72-2.16) |
| Alpha variant  (February 1 - June 30, 2021) | 4.88 (1.67-14.24) | 2.07 (1.14-3.76) | | 1.42 (0.76-2.68) | 1.85 (1.01-3.36) |
| Omicron variant  (January 1 - May 31, 2022) | 9.25 (3.30-25.92) | 2.59 (1.50-4.48) | | 2.59 (1.45-4.65) | 2.55 (1.46-4.44) |
| Age |  |  |  |  |  |
| <30 years |  |  | |  |  |
| 30-34 years |  |  | |  |  |
| ≥35 years |  |  | |  |  |
| Citizenship |  |  |  |  |  |
| Not Italian |  |  | |  | 1 |
| Italian |  |  | |  | 0.67 (0.46-0.97) |
| Level of education |  |  |  |  |  |
| Low |  |  | |  | 0.44 (0.27-0.74) |
| Medium |  |  | |  | 0.66 (0.42-1.05) |
| High |  |  | |  | 1 |
| Parity |  |  |  |  |  |
| Nulliparous |  |  | |  |  |
| Multiparous |  |  | |  |  |
| Gestational age at birth |  |  |  |  |  |
| <37 weeks | 0.37 (0.24-0.57) | 0.23 (0.16-0.32) | | 0.10 (0.07-0.14) | 0.32 (0.23-0.46) |
| ≥37 weeks | 1 | 1 | | 1 | 1 |
| COVID-19 pneumonia |  |  |  |  |  |
| No | 1 | 1 | | 1 | 1 |
| Yes | 0.49 (0.24-0.99) | 0.29 (0.16-0.51) | | 0.20 (0.12-0.35) | 0.35 (0.21-0.56) |
| Volume of deliveries of maternity units |  |  |  |  |  |
| <1,000 | 1 |  | | 1 | 1 |
| 1,000-1,999 | 0.94 (0.69-1.28) |  | | 1.35 (0.95-1.91) | 2.05 (1.39-3.01) |
| ≥2,000 | 0.44 (0.31-0.61) |  | | 0.75 (0.53-1.06) | 0.72 (0.49-1.06) |
| Geographical location of maternity units |  |  |  |  |  |
| North Italy | 1 | 1 | | 1 | 1 |
| Centre Italy | 0.61 (0.42-0.88) | 0.84 (0.61-1.15) | | 0.18 (0.12-0.27) | 0.35 (0.23-0.54) |
| South Italy | 0.31 (0.22-0.42) | 0.25 (0.19-0.33) | | 0.04 (0.03-0.05) | 0.10 (0.07-0.14) |

In the models performed by stepwise procedure, the following variables were considered: pandemic phase, age, citizenship, level of education, parity, gestational age at birth, COVID-19 pneumonia, volume of deliveries of maternity units, geographical location of maternity units.

**Table S3a** Mutually adjusted odds ratios for the reported variables among women who had a vaginal birth. Logistic regression models on complete cases.

|  | **Presence of a labour companion (Yes *vs* No)** | **Skin-to-skin**  **(Yes *vs* No)** | **No mother-child separation at birth (Yes *vs* No)** | **Rooming-in (Yes *vs* No)** | **Breastfeeding**  **(Yes *vs* No)** | | | |
| --- | --- | --- | --- | --- | --- | --- | --- | --- |
|  | **OR (95% CI)** | **OR (95% CI)** | **OR (95% CI)** | **OR (95% CI)** | **OR (95% CI)** | | | |
| Pandemic phase |  |  |  |  |  | | | |
| Wild-type virus, phase 1  (25 February - May 31, 2020) | 1 | 1 | 1 | 1 | 1 | | | |
| Wild-type virus, phase 2  (June 1 - August 31, 2020) | 1.52 (0.87-2.67) | 3.84 (1.85-7.96) | 1.61 (0.81-3.20) | 1.22 (0.48-3.11) | 2.35 (0.57-9.61) | | | |
| Wild-type virus, phase 3  (September 1, 2020 – January 31, 2021) | 0.69 (0.48-0.99) | 5.37 (3.30-8.72) | 1.08 (0.75-1.56) | 1.17 (0.69-2.01) | 1.43 (0.72-2.82) | | | |
| Alpha variant  (February 1 - June 30, 2021) | 0.83 (0.56-1.23) | 11.23 (6.55-19.26) | 1.60 (1.05-2.43) | 1.76 (0.97-3.19) | 2.15 (0.99-4.67) | | | |
| Omicron variant  (January 1 - May 31, 2022) | 1.37 (0.97-1.95) | 18.52 (11.34-30.25) | 2.12 (1.46-3.07) | 2.49 (1.44-4.30) | 2.94 (1.48-5.84) | | | |
| Age |  |  |  | | |  |  |  |
| <30 years |  |  |  |  |  | | | |
| 30-34 years |  |  |  |  |  | | | |
| ≥35 years |  |  |  |  |  | | | |
| Citizenship |  |  |  | | |  |  |  |
| Not Italian | 1 |  |  |  | 1 | | | |
| Italian | 1.46 (1.22-1.73) |  |  |  | 0.54 (0.36-0.81) | | | |
| Level of education |  |  |  | | |  |  |  |
| Low |  | 0.69 (0.52-0.90) |  |  | 0.56 (0.37-0.85) | | | |
| Medium |  | 0.74 (0.57-0.95) |  |  | 0.78 (0.54-1.15) | | | |
| High |  | 1 |  |  | 1 | | | |
| Parity |  |  |  | | |  |  |  |
| Nulliparous |  | 0.81 (0.67-0.99) |  |  |  | | | |
| Multiparous |  | 1 |  |  |  | | | |
| Gestational age at birth |  |  |  | | |  |  |  |
| <37 weeks |  | 0.25 (0.17-0.39) | 0.22 (0.15-0.32) | 0.13 (0.09-0.20) | 0.33 (0.20-0.56) | | | |
| ≥37 weeks |  | 1 | 1 | 1 | 1 | | | |
| COVID-19 pneumonia |  |  |  | | |  |  |  |
| No |  | 1 | 1 | 1 |  | | | |
| Yes |  | 0.51 (0.28-0.93) | 0.31 (0.20-0.48) | 0.44 (0.24-0.81) |  | | | |
| Volume of deliveries of maternity units |  |  |  | | |  |  |  |
| <1,000 | 1 | 1 |  | 1 | 1 | | | |
| 1,000-1,999 | 1.19 (0.98-1.45) | 0.78 (0.61-1.01) |  | 1.22 (0.91-1.62) | 1.67 (1.15-2.43) | | | |
| ≥2,000 | 0.82 (0.67-0.99) | 0.50 (0.38-0.65) |  | 0.64 (0.48-0.86) | 0.49 (0.34-0.71) | | | |
| Geographical location of maternity units |  |  |  | | |  |  |  |
| North Italy | 1 | 1 | 1 | 1 | 1 | | | |
| Centre Italy | 0.39 (0.31-0.49) | 0.48 (0.36-0.63) | 0.42 (0.33-0.53) | 0.16 (0.12-0.22) | 0.29 (0.19-0.45) | | | |
| South Italy | 0.18 (0.14-0.24) | 0.14 (0.11-0.18) | 0.11 (0.09-0.13) | 0.03 (0.02-0.04) | 0.06 (0.04-0.09) | | | |

In the models performed by stepwise procedure, the following variables were considered: pandemic phase, age, citizenship, level of education, parity, gestational age at birth, COVID-19 pneumonia, volume of deliveries of maternity units, geographical location of maternity units.

**Table S3b** Mutually adjusted odds ratios for the reported variables among women who underwent caesarean section. Logistic regression models on complete cases.

|  | **Skin-to-skin**  **(Yes *vs* No)** | **No mother-child separation at birth (Yes *vs* No)** | | **Rooming-in (Yes *vs* No)** | **Breastfeeding**  **(Yes *vs* No)** |
| --- | --- | --- | --- | --- | --- |
|  | **OR (95% CI)** | **OR (95% CI)** | | **OR (95% CI)** | **OR (95% CI)** |
| Pandemic phase |  |  | |  | |
| Wild-type virus, phase 1  (25 February - May 31, 2020) | 1 | 1 | | 1 | 1 |
| Wild-type virus, phase 2  (June 1 - August 31, 2020) | 3.31 (0.76-14.43) | 1.24 (0.50-3.06) | | 1.58 (0.54-4.64) | 2.30 (0.59-8.97) |
| Wild-type virus, phase 3  (September 1, 2020 – January 31, 2021) | 4.61 (1.39-15.27) | 1.74 (0.98-3.09) | | 1.32 (0.67-2.61) | 1.20 (0.57-2.49) |
| Alpha variant  (February 1 - June 30, 2021) | 4.64 (1.34-16.01) | 2.31 (1.24-4.29) | | 1.42 (0.68-2.96) | 1.95 (0.87-4.36) |
| Omicron variant  (January 1 - May 31, 2022) | 10.40 (3.17-34.15) | 2.70 (1.53-4.76) | | 2.63 (1.33-5.20) | 2.75 (1.31-5.79) |
| Age |  |  |  |  |  |
| <30 years |  |  | |  |  |
| 30-34 years |  |  | |  |  |
| ≥35 years |  |  | |  |  |
| Citizenship |  |  |  |  |  |
| Not Italian |  |  | |  | 1 |
| Italian |  |  | |  | 0.60 (0.39-0.93) |
| Level of education |  |  |  |  |  |
| Low |  |  | |  | 0.42 (0.26-0.66) |
| Medium |  |  | |  | 0.62 (0.41-0.92) |
| High |  |  | |  | 1 |
| Parity |  |  |  |  |  |
| Nulliparous |  |  | |  |  |
| Multiparous |  |  | |  |  |
| Gestational age at birth |  |  |  |  |  |
| <37 weeks | 0.32 (0.21-0.48) | 0.20 (0.14-0.29) | | 0.09 (0.06-0.13) | 0.32 (0.22-0.48) |
| ≥37 weeks | 1 | 1 | | 1 | 1 |
| COVID-19 pneumonia |  |  |  |  |  |
| No |  | 1 | | 1 | 1 |
| Yes |  | 0.28 (0.16-0.51) | | 0.20 (0.11-0.37) | 0.54 (0.31-0.94) |
| Volume of deliveries of maternity units |  |  |  |  |  |
| <1,000 | 1 |  | | 1 | 1 |
| 1,000-1,999 | 0.88 (0.64-1.21) |  | | 1.26 (0.88-1.79) | 1.50 (0.99-2.26) |
| ≥2,000 | 0.43 (0.31-0.61) |  | | 0.67 (0.47-0.96) | 0.67 (0.44-1.02) |
| Geographical location of maternity units |  |  |  |  |  |
| North Italy | 1 | 1 | | 1 | 1 |
| Centre Italy | 0.60 (0.42-0.88) | 0.82 (0.59-1.12) | | 0.17 (0.12-0.26) | 0.40 (0.25-0.64) |
| South Italy | 0.29 (0.21-0.41) | 0.24 (0.18-0.33) | | 0.04 (0.03-0.05) | 0.14 (0.09-0.20) |

In the models performed by stepwise procedure, the following variables were considered: pandemic phase, age, citizenship, level of education, parity, gestational age at birth, COVID-19 pneumonia, volume of deliveries of maternity units, geographical location of maternity units.
